# Molecular epidemiology of carbapenem-resistant hypervirulent *Klebsiella pneumoniae* in China

**DOI:** 10.1080/22221751.2022.2049458

**Published:** 2022-03-19

**Authors:** Xuemei Yang, Qiaoling Sun, Jiaping Li, Yu Jiang, Yi Li, Jianping Lin, Kaichao Chen, Edward Wai-Chi Chan, Rong Zhang, Sheng Chen

**Affiliations:** a Department of Infectious Diseases and Public Health, City University of Hong Kong, Kowloon, Hong Kong; b Department of Clinical Laboratory Medicine, Second Affiliated Hospital of Zhejiang University, Hangzhou, People’s Republic of China; c Department of Clinical Laboratory Medicine, Frist Affiliated Hospital of Zhejiang University, Hangzhou, People’s Republic of China; d Department of Clinical Laboratory Medicine, Henan Provincial People’s Hospital, Zhengzhou, People’s Republic of China; e Department of Clinical Laboratory Medicine, Wenzhou people’s Hospital, Wenzhou, People’s Republic of China; f State Key Laboratory of Chirosciences, Department of Applied Biology and Chemical Technology, The Hong Kong Polytechnic University, Kowloon, Hong Kong

**Keywords:** Klebsiella pneumoniae, CR-HvKP, prevalence, epidemiology, virulence plasmid

## Abstract

The epidemiological features of the newly emerged carbapenem-resistant hypervirulent *Klebsiella pneumoniae* (CR-HvKP) and its potential threat to human health are currently unknown. In this study, a total of 784 *bla*_KPC-2_-bearing CRKP strains collected from three hospitals located at different geographical locales in China during 2014–2017 were subjected to molecular typing, screening of virulence plasmid, string test and WGS (367/784 strains). The proportion of CRKP among all clinical *K. pneumoniae* strains increased sharply in China during 2014–2017. A large proportion (58%) of these CRKP strains were found to harbour a virulence-encoding plasmid, while only 13% of such strains exhibited a hypervirulent phenotype by string test and neutrophil assay. The lack of hypervirulent phenotype in virulent plasmid-bearing CRKP strains was found to be due to the mutation’s presence on *rmpA* and *rmpA2* genes, which rendered them non-functional, while some strains carrying wild type *rmpA* did not exhibit hypervirulent phenotype either suggesting that other factors might also contribute to the hypervirulence of CRKP. Phylogenetic and SNP analysis indicated that the transmission of these CRKP strains in China likely involved several major clones of ST11. Carriage of IncFII pSWU01-like, *bla*_KPC-2_-bearing plasmid was found to be the major mechanism of carbapenem resistance in these CRKP strains. In conclusion, our data indicated that the prevalence of CRKP strains carrying the virulence plasmid has rapidly increased in China, while genetic markers were not correlated well with the hypervirulent phenotypes, which call for a better definition and screening for these truly hypervirulent CR-HvKP strains in clinical settings.

## Introduction

Despite being a member of the human gut microflora, *Klebsiella pneumoniae* has evolved into several strains that encode different resistance and virulence phenotypes, imposing an enormous threat to human health. Upon acquisition of a plasmid which harbours a carbapenemase gene, it becomes carbapenem-resistant *K. pneumoniae* (CRKP), a superbug which causes untreatable or hard-to-treat infections, and is therefore considered an urgent threat to public health by the US CDC [1,2]. CRKP strains constitute a worldwide problem, especially in endemic areas [3]. Since the first report of emergence of CRKP strains in China in 2007 [4], it has been increasingly recoverable in clinical settings. In 2016, the prevalence of CRKP ranged from 0.9% to 23.6% in different provinces in China, with an average rate of 8.7% [5]. Apart from antimicrobial resistance, another worrisome development is related to the evolution of hypervirulent *Klebsiella pneumoniae* (HvKP). First recovered from patients with liver abscess in Taiwan in late 1980s, HvKP causes life-threatening, community and hospital-acquired infections such as liver abscess, pneumonia, meningitis and endophthalmitis, often in relatively young and healthy individuals. Associated with a high morbidity and mortality rate [6], these strains are able to acquire iron efficiently and produce an increased amount of capsular substance, which confers a hypermucoviscous phenotype detectable as a positive “string test” result [6]. This hypermucoviscous phenotype of HvKP was thought to be attributed to the carriage of a virulence plasmid which harbours two CPS regulator genes (*rmpA* and *rmpA2*) and a number of siderophore gene clusters [7,8]. Ever since their emergence, CRKP has been an organism of relatively low virulence, whereas HvKP remains antibiotic-sensitive until recently. Our laboratory reported, in 2015, that HvKP strains had evolved to become carbapenem-resistant K1 hypervirulent (HvKP) organisms through the acquisition of a carbapenemase-encoding plasmid, causing fatal infections among patients without liver abscess [9]. A further ominous sign was observed in 2017, when we isolated ST11 carbapenem-resistant HvKP strains (ST11 CR-HvKP) for the first time from patients with fatal pulmonary infections [10]. ST11 CR-HvKP was found to have evolved from ST11 CRKP by the acquisition of a virulence plasmid originally harboured by HvKP. ST11 CRKP belongs to the same clonal group as ST258 and is the most dominant clone in Asia, accounting for over 60% of clinical CRKP strains in China in 2015 [11–13]. Such strain is therefore considered the most transmissible clone that contributes to the continuous increase in the prevalence of CRKP in China in recent years. Taken together, ST11 CR-HvKP is considered a real superbug which exhibits high transmissibility, hyper-resistance and hypervirulence, posing a grave threat to human health. Such strain has since been detectable worldwide, but China remains the country of the highest prevalence [9,14–19]. Likewise, analysis of available genome sequences in the GenBank also confirms that CRKP carrying the virulence plasmid have insidiously disseminated to other parts of the world (data not shown). This scenario prompts immediate action to halt transmission of this dangerous organism.

## Materials and methods

### Sources of bacterial strains

CRKP strains collected from all clinical departments of the three participating hospitals during the period of 2014–2017 were purified for further analysis. Second Affiliated Hospital of Zhejiang University (SAHZU) and Wenzhou Tertiary Hospital (WZTH), are located in different cities of the same province, Zhejiang province, China. The third hospital, Henan Provincial People’s Hospital (HPPH), is located in a city in Henan province, which is geographically distant from Zhejiang Province. SAHZU is located in Hangzhou city, contains 3500 beds and serves mainly Hangzhou city (population of 9 million). WZTH is located in Wenzhou city of Zhejiang Province, contains 2500 beds and serves mainly Wenzhou city (population of over 9 million). HPPH is located in Henan province, contains 3900 beds and serves mainly Zhengzhou city in Henan province (population of 9 million). Each of these three hospitals serves the city as well as a wide catchment area covering neighbouring towns.

### Determination of minimal inhibitory concentration (MICs)

All CRKP strains were cultured on Columbia blood agar (Oxoid, Hampshire, UK) containing 5% sheep blood (Luqiao, Beijing, China). Strain identity was confirmed by MALDI-TOF mass spectrometry (BrukerDaltonik GmbH, Bremen, Germany). Antimicrobial susceptibilities were determined by the VITEK-2 compact system (bioMérieux) and interpreted according to the guideline document M100-S31 established by the Clinical and Laboratory Standards Institute (CLSI) [20]. Susceptibilities to colistin and tigecycline were determined by the broth dilution method and interpreted according to the European Committee on Antimicrobial Susceptibility Testing (EUCAST) criteria (available at http://www.eucast.org/clinical_breakpoints/).

### Genetic and phenotypic characterization

To determine the prevalence of carbapenem-resistant hypervirulent *K. pneumoniae* (CR-HvKP) in clinical settings in these three hospitals, all the 784 *bla*_KPC-2_-bearing CRKP strains were subjected to screening of several genetic markers located in the virulence plasmid including *rmpA*, *rmpA2* and *iutA* as previously described [10]. In addition, all test strains were subjected to the string test as described previously to determine if they expressed the hypermucoviscous phenotype [10]. All strains with positive string test phenotype were subjected to neutrophil survival assay. Strains that further exhibited a positive result of the neutrophil survival assay was defined as phenotypic hypervirulence [10]. Multi locus sequence typing (MLST) was performed on all CRKP strains as previously described [21]. Screening of virulence plasmid, string test and neutrophil survival assays was performed as previously described [6,10].

### Whole genome sequencing and data analysis

Genomic DNA was extracted and sequenced via the NextSeq 500 sequencing platform (Illumina, San Diego, CA, USA). Single nucleotide polymorphisms (SNPs) were identified via mapping of Illumina raw reads to a reference genome (*Klebsiella pneumoniae* subsp. pneumoniae HS11286, NC_016845.1). An alignment of “core SNPs” was produced using Snippy and used to build a high-resolution phylogeny [22]. Lineages were defined based on patristic distances in the ML tree using FastTree [23]. The output tree was then merged to attain a dated tree with the online TreeAnnotator software iTOL (http://itol.embl.de/) [24]. De novo assemblies of Illumina reads were generated using SPAdes Genome Assembler version 3.11.1 [25]. Assembled draft genome sequences were annotated with RAST v2.0 [26] and Prokka v1.14.5 [27]. The sequence types (STs) were determined by the Kleborate software based on genetic variation in the seven housekeeping genes [28]. Capsular typing on the assembled sequences were performed using Kaptive [29]. Virulence genes were identified by matching against the BIGSdb Klebsiella genome database [30]. The BLAST command lines, with an 80% coverage cutoff, were used to map genome sequences against antibiotic resistance genes and plasmid replicons. The resistance genes and plasmid replicons databases were obtained from the Center for Genomic Epidemiology [31]. To check for the presence of the virulence plasmid, genome sequences were compared to a reference plasmid pLVPK [8] using the BLAST Ring Image Generator (BRIG) version 0.95.22 [32].

## Results

### Prevalence of CRKP and virulence plasmid-bearing CRKP strains

The prevalence of CRKP strains in three participating hospitals during the period 2014–2017 was first determined. The number of clinical *K. pneumoniae* strains isolated in each of the three hospitals was found to stably increase during this period; likewise, the rate of CRKP infection also increased from 20% in 2014 to 36.3% in 2017 in SAHZU, from 8.7% in 2014 to 61.4% in 2017 in HPPH, and from 6.4% in 2014 to 18% in 2017 in WZTH (Table S1).

The epidemiological features of the CRKP strains were further investigated. CRKP strains collected from all clinical departments of the three participating hospitals during the period of 2014–2017 were purified and retrospective analysis of clinical data of patients from whom the CRKP strains were recovered was performed. Only one CRKP strain collected from each of the patients with a complete clinical dataset was selected for further study, resulting in 784 CRKP strains. Screening of carbapenemase genes indicated that these 784 CRKP strains were all positive for *bla*_KPC-2_. To assess the prevalence of pLVPK-like virulence plasmids among these *bla*_KPC-2_-bearing CRKP strains, we performed screening of the conservative, plasmid-borne virulence genes *rmpA*, *rmpA2* and *iutA* and found that carriage of the virulence plasmid among clinical CRKP strains was a common event, with 457/784 (58%) of the CRKP strains tested carrying at least two of these three genes. The overall carriage rate of the virulence plasmid in CRKP strains collected from these three hospitals increased dramatically from 2014 to 2016, dropping slightly in SAHZU and HPPH in 2017, whereas the carriage rate in WZTH declined gradually over the years (Table 1). String test was also performed on all 784 strains regardless of their status of carriage of virulence plasmid. Overall 64 (8%) out of the 784 *bla*_KPC-2_-bearing CRKP strains were positive for string test. All the string test positive strains carried a pLVPK-like virulence plasmid, while the carriage of the virulence plasmid did not correlate with the string test results. Only 13% of the 457 clinical CRKP strains that harboured the virulence plasmid were positive for string test; 60 of these 64 strains exhibited high survival rate (>70%) in neutrophils. We define these 60 strains that carried *rmpA2* and *iutA*, and exhibited hypermucovisity phenotype and >70% survival rate in the neutrophil assay as phenotypically hypervirulent (phenotypic CR-HvKP). It should be noted that in one hospital (SAHZU), the recovery rate of phenotypic CR-HvKP increased from none in 2014 and 2015–6% and 18% in 2016 and 2017 respectively (Table 1).

**Table 1.** Prevalence of CRKP and virulence plasmid-bearing CRKP (including both non-phenotypic CR-HvKP and phenotypic CR-HvKP) strains in three different hospitals in China; the proportion of ST11 type among these strains is also shown.

Hospital	Year	Number of strains (%)				
		CRKP	Virulence plasmid-bearing CRKP	phenotypic CR-HvKP	Total	ST11
HPPH	2014	16 (53)	14 (47)	3 (10)	30	21 (70)
	2015	12 (44)	15 (56)	2 (7)	27	23 (85)
	2016	33 (34)	65 (66)	3 (3)	98	91 (93)
	2017	17 (43)	12 (57)	3 (4)	29	27 (93)
	Total	78 (42)	106 (58)	11 (6)	184	162 (88)
SAHZU	2014	34 (90)	4 (11)	0	38	33 (87)
	2015	69 (76)	21 (23)	0	90	76 (84)
	2016	12 (12)	87 (88)	6 (6)	99	91(92)
	2017	29 (29)	70 (71)	18 (18)	99	70 (71)
	Total	150 (46)	176 (54)	24 (7)	326	272 (83)
WZTH	2014	21 (28)	53 (72)	7 (10)	74	71 (96)
	2015	25 (30)	58 (70)	9 (11)	83	63 (76)
	2016	10 (31)	22 (69)	2 (6)	32	31 (97)
	2017	49 (58)	36 (42)	7 (8)	85	77 (91)
	Total	105 (38)	169 (62)	25 (9)	274	242 (88)
All three hospitals	2014	71 (50)	71 (50)	10 (7)	142	125 (88)
	2015	106 (53)	94 (47)	11 (6)	200	162 (81)
	2016	55 (24)	174 (76)	11 (5)	229	213 (93)
	2017	95 (45)	118 (55)	28 (13)	213	174 (82)
	Total	327 (42)	457 (58)	60 (8)	784	674 (86)

### MIC profiles of CRKP and CR-HvKP

Almost all CRKP strains exhibited resistance to commonly used antibiotics such as cephalosporins, carbapenems and fluoroquinolone, although a slightly lower rate of resistance to amikacin (73%) was observable. On the other hand, however, the majority of these strains exhibited susceptibility to colistin (96%) and tigecycline (76%). Antibiotic susceptibility profiles of CR-HvKP were similar to those of CRKP, with high susceptibility to colistin and tigecycline being observable. Nevertheless, 7% (4/60) of phenotypic CR-HvKP strains have become resistant to colistin, suggesting further evolution of these hyper-resistant and hypervirulent strains. No *mcr-1* gene was detectable in these colistin-resistant CR-HvKP strains; the colistin resistance in these strains was due to the inactivation of the *mgrB* gene by IS*kpn18* element (data not shown). Screening of carbapenemase genes showed that all test strains carried *bla*_KPC-2_, the most common cabapenemase gene detectable among clinical carbapenem-resistant *Enterobacteriaceae* strains; none of the strains were found to harbour other carbapenemase genes (Table 2).

**Table 2.** Antimicrobial susceptibility of clinical CRKP and CR-HvKP strains.

Antibiotics	Range	CRKP (*n* = 784)				CR-HvKP (*n* = 60)			
		MIC_50_	MIC_90_	%S	%R	MIC_50_	MIC_90_	%S	%R
Ceftazidime	≤4–>128	64	>128	1	99	32	>128	0	100
Cefotaxime	≤4–>128	>128	>128	0	100	>128	>128	0	100
Amikacin	≤4–>128	>128	>128	27	73	>128	>128	18	82
Ciprofloxacin	≤1–>32	>32	>32	2	98	32	>32	0	100
Cefmetazole	≤2–>128	128	>128	2	98	128	>128	0	100
Cefepime	≤4–>64	>64	>64	1	99	>64	>64	0	100
Piperacillin- tazobactam	128/4–>256/4	>256/4	>256/4	0	100	>256/4	>256/4	0	100
Colistin	≤0.5–>8	≤0.5	2	96	4	≤0.5	1	94	6
Imipenem	≤0.5–>128	32	64	1	100	32	64	0	100
Meropenem	2–>128	128	>128	0	100	128	>128	0	100
Ertapenem	8–>128	>128	>128	0	100	128	>128	0	100
Cefoperazone- sulbactam	32/16–>128/64	>128/64	>128/64	0	100	>128/64	>128/64	0	100
Tigecycline^a^	≤0.25–4	1	2	79	21	1	2	69	31
Aztreonam	32–>128	>128	>128	0	100	>128	>128	0	100

^a^ For tigecycline, NS (non-susceptible) % was used here to replace R% (resistance). The proportion of CRKP and CR-HvKP strains that exhibited intermediate resistance was 19% and 31%, respectively.

### Increasing prevalence of ST11 type among CRKP and CR-HvKP

**Figure 1.**
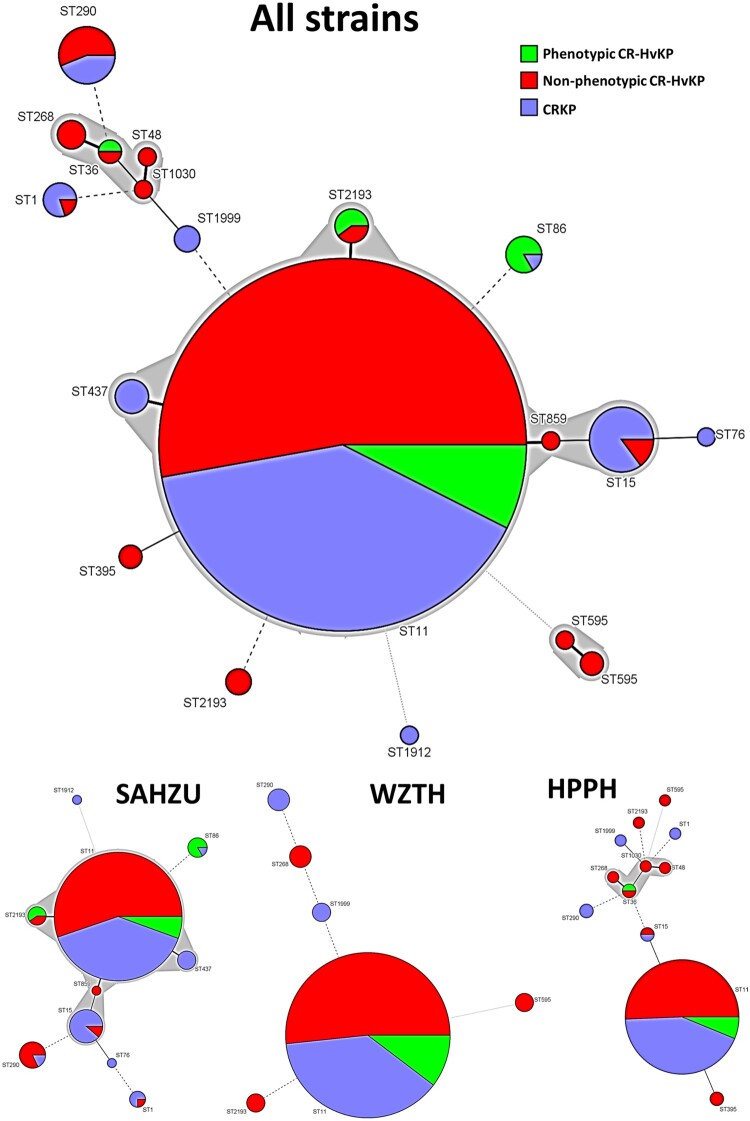
ST distribution among CRKP, non-phenotypic and phenotypic CR-HvKP strains. ST11 (*n* = 676), ST15 (*n* = 20), ST290 (*n* = 16), ST2193 (*n* = 8), ST86 (*n* = 6), ST1 (*n* = 5), ST437 (*n* = 5), ST268 (*n* = 4), ST595 (*n* = 3), ST395 (*n* = 2), ST48, ST76, ST859, ST1030, ST1912 (*n* = 1). SAHZU, Second Affiliated Hospital of Zhejiang University; HPPH, Henan Province People’s Hospital; WZTH, Wenzhou Tertiary Hospital.

Among the 784 *bla*_KPC-2_-bearing CRKP strains tested, 29 were found to belong to new ST types, whereas the others fell into 17 different ST types. ST11 was the most common, accounting for 676/784 (86%) of the test strains, followed by ST15 (20), ST290 (16), ST2193 (8) and ST86 (6). Other ST types appear to cluster at specific hospitals (Figure 1). In HPPH, the proportion of ST11 among all CRKP and CR-HvKP strains increased from 70% in 2014 to 93% in 2016 and 2017. In SAHZU and WZTH, the proportion of ST11 among all CRKP and CR-HvKP strains was 83% and 88% respectively on average during the period 2014 through 2017 (Table 1). For CRKP strains carrying the virulence plasmid, 407/457 (88%) belonged to ST11. Among the 60 phenotypic CR-HvKP strains, the majority (*n* = 50 or 83%) also belonged to ST11 type (Figure 1).

### Molecular epidemiology of CRKP strains in three hospitals in China

**Figure 2.**
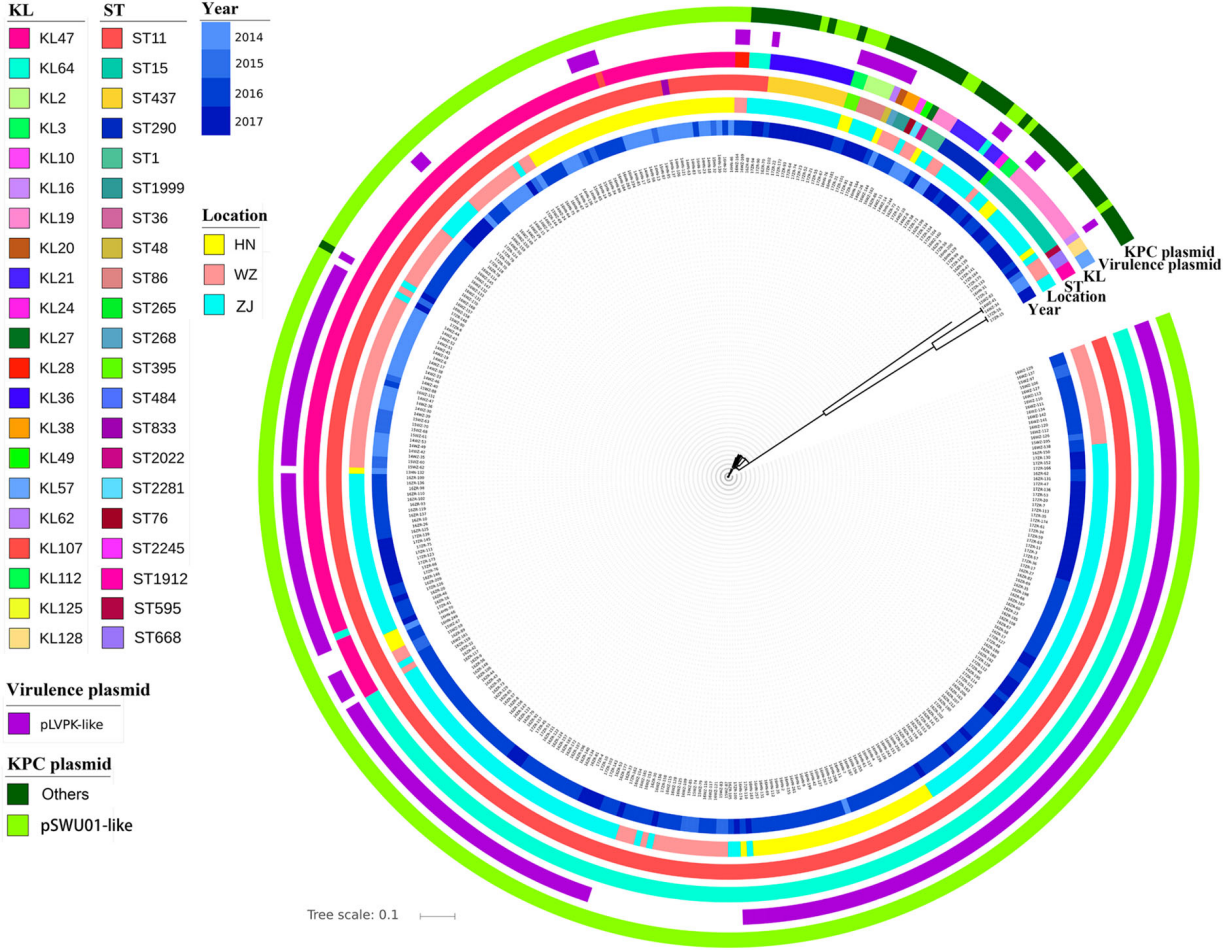
Genetic characteristics of CRKP strains. KL, serotypes; ST, ST types; Locations: ZJ (SAHZU), Second Affiliated Hospital of Zhejiang University; HN (HPPH), Henan Province People’s Hospital; WZ (WZTH), Wenzhou Tertiary Hospital. The *bla*_KPC-2_-bearing plasmid: IncFII – carriage of IncFII replicon; non-IncFII – without carriage of IncFII replicon.

To obtain deeper understanding of these strains at the molecular level, 367 of the 784 test strains were randomly selected for whole genome sequencing (WGS), including 192 from SAHZU, 77 from HPPH and 98 from WZTH, among which 238 were found to harbour the virulence plasmid whereas 129 did not. The sequence data of these strains were consistent with the PCR screening results in terms of ST typing and carriage of virulence plasmids. Phylogenetic analysis showed that, regardless of status of carriage of virulence plasmid, the test strains were clustered according to ST types and KL-types (Figure 2). For ST11 type strains, four KL-types were detectable with KL47 and KL64 being the most dominant in each of the three hospitals, whereas KL28 and KL117 were only detectable in SAHZU and WETH respectively (Figure S1, Table S2). Detection of these four KL-types allows further differentiation of the ST11 CRKP strains into four different clades (Figure S1). Among the KL47 and KL64 types of CRKP, strains from the same hospital seemed to be clustered together (Figure S1). SNPs analysis showed that ST11 CRKP strains of the same KL-type and from the same hospital often exhibited SNPs of <10, suggesting that these strains belonged to the same clone and that they were transmissible within the hospital (Table S3). Strains from different hospitals also exhibited SNPs of <100, suggesting that these ST11 strains might be derived from the same ancestor and have disseminated to different hospitals, where they kept evolving (Table S3).

### Molecular epidemiology of pLVPK like virulence plasmid and *bla*_KPC-2_-bearing plasmid in these CRKP strains

Total 238 sequenced *bla*_KPC-2_-bearing CRKP strains that were positive for *rmpA*, *rmpA2* or *iuc* were determined as pLVPK-positive (Table S2). Genome sequences of these 238 strains were aligned using pLVPK as a reference by BRIG. All the strains carried different profiles of DNA segments from pLVPK, suggesting that they were derived from a separate origin (Figure S2). Interestingly, plasmids recovered from strains in the same hospital often displayed a unique sequence profile, with plasmids from SAHZU and WZTH exhibiting closer genetic relationship than those obtained from HPPH, suggesting that clonal spread of CRKP occurred in the hospital. It should be noted that virulence plasmids recovered from strains in the same hospital were also found to have undergone evolution, resulting in the emergence of plasmids of different profiles (Figure S2). To investigate the molecular basis of inconsistency between carriage of virulence plasmid and string test results, we aligned the nucleotide sequences of the *rmpA* and *rmpA2* genes of the test strains to the two genes in pLVPK. Our data showed that the *rmpA2* gene in virulence plasmids obtained from strains that produced negative string test results contained mutations at different sites, with a total of 9 mutation types being identified *rmpA2* gene. The *rmpA* gene in the majority of the test strains also exhibited mutations at different sites, with a total of 5 mutation types being identified (Figure S3, Table S2). On the other hand, all the 30 phenotypic CR-HvKP strains sequenced were found to harbour virulence plasmid which contained the WT sequence of *rmpA/rmpA2 mutation* (*n* = 16) or the variants of *rmpA*_M5 (*n* = 5)/*rmpA2* mutations, *rmpA2*_M1 (*n* = 6), *rmpA2*_M3 (*n* = 1), *rmpA2*_M5 (*n* = 1), *rmpA2*_M9 (*n* = 1). Among them, *rmpA*_M5, *rmpA2_*M3, *rmpA2_*M5 and *rmpA2_*M9 were known to contain deletion or insertion mutations, implying that mechanisms other than *rmpA/rmpA2* could also mediate hypermucoviscous phenotype. The hypermucoviscosity phenotype can also be mediated by the genes associated with capsule biosynthesis [33]. The capsule biosynthesis locus of all the sequenced strains have been annotated (Table S2), however, the comparison of strains harbouring mutated *rmpA/rmpA2* didn’t identify specific determinants. On the other hand, we also notice that some strains carrying the above WT sequences of *rmpA* or *rmpA/rmpA2* mutations did exhibit the hypervirulence phenotype, suggesting that genetic traits other than *rmpA/rmpA2* also play a role in regulating the expression of the hypervirulence phenotype. SNP analysis was also performed on the 30 sequenced phenotypic CR-HvKP strains, including the five outbreak CR-HvKP strains reported in an earlier study [2], with results showing that these ST11 CR-HvKP belonged to two genetically distinct clones (Figure S4, Table S4).

Plasmids carrying the *bla*_KPC-2_ gene in all 367 strains which were randomly selected from the 784 *bla*_KPC-2_-bearing CRKP strains with WGS were analyzed by sequence alignment to a reference plasmid, pSWU01 (NZ_CP018455.1), a *bla*_KPC-2_-bearing plasmid recovered from a clinical *K. pneumoniae* strain SWU01 in Sichuan Province, China. Our data showed that 321 of these 367 CRKP strains, including all ST11 type strains regardless of whether they harboured a virulence plasmid, carried a plasmid of IncFII replicon, which aligned to pSWU01 with 32-96% coverage (Figure 2, Table S2). Among the 58/367 non-ST11 type CRKP strains with WGS, 17 and 41 were found to harbour the IncFII plasmid and a non IncFII plasmid respectively (Figure 2, Table S2). The data suggested that the pSWU01-like plasmid played a significant role in the transmission of carbapenem resistance in these *bla*_KPC-2_-bearing CRKP strains. Alignment of the pSWU01-like plasmid obtained from CRKP strains in different hospitals showed that plasmids which originated from different hospital exhibited different patterns, thereby further suggesting that pSWU01-like plasmids also underwent genetic rearrangement in the same hospital (Figure S5).

## Discussion

In this study, we investigated molecular epidemiology of clinically isolated carbapenem-resistant hypervirulent *K. pneumoniae* (CR-HvKP), a real and newly emerged superbug that comprises the genetic features of both CRKP and HvKP. Our data show that the rate of carriage of virulence plasmid in the *bla*_KPC-2_-bearing CRKP in the three participating hospitals increased significantly over the past few years, with 58% of the *bla*_KPC-2_-bearing CRKP strains carrying a virulence plasmid. Other important observations in this work are that ST11 type of strains is dominant among these *bla*_KPC-2_-bearing CRKP strains, with 88% of these *bla*_KPC-2_-bearing CRKP strains belonging to this sequence type, and that the proportion of ST11 strains that carry virulence plasmid is much higher than other ST types. In the three hospitals, ST11 is extremely rare among non-CRKP strains (data not shown). Such discrepancy suggests that ST11 CRKP strains were most likely responsible for acquisition and dissemination of the virulence plasmid. Phylogenetic analysis indicated that ST11 CRKP/ CR-HvKP strains could be grouped into four clades, with two being dominant. In the same hospital, CRKP strains from the same genetic clade were shown to belong to the same clone, suggesting that clonal spread of CRKP occurred in the hospital. Strains recovered from different hospitals also exhibited close genetic linkage (less than 100 SNPs), suggesting that they might originate from the same clone and spread to different parts of China. Virulence plasmids and *bla*_KPC-2_-bearing plasmids recovered from CRKP strains in one hospital were found to exhibit unique genetic pattern which differed from those recovered from the other two hospitals, indicating that the virulence plasmid continued to evolve within the hospital. Likewise, it should be noted that CRKP strains also rapidly evolve within the clinical setting, as our data show that other ST types of CRKP also begin to harbour the virulence plasmid, and that such strains have become increasingly prevalent. Consistent with these findings, several recent studies also reported that CR-HvKP has disseminated extensively in various parts of China as well as other countries, causing extremely high mortality among infected patients [9,14–19]. Further works are required to depict the genetic events that enable the virulence plasmid to be stably harboured by specific host organisms.

An increasing body of evidence shows that carriage of virulence plasmid does not necessarily correlate with expression of the hypermucoviscous phenotype of *K. pneumoniae*, and that detection of the hypermucoviscous phenotype may not correlate with expression of the hypervirulence phenotype of this organism [34]. Our molecular epidemiological data showed that a large proportion of CRKP strains carried virulence plasmids, the majority of which exhibited over 80% similarity to pLVPK. Some of these plasmids, resembling pLVPK, carry both the *rmpA* and *rmpA2* genes, whereas others carry only the *rmpA2* gene, in a manner similar to the plasmid pVir-CRHVKP2, which was recently reported in China [2,35]. It should be noted that the *rmpA2* gene in these plasmids is often structurally different from the WT *rmpA2* gene by harbouring various mutations or insertions/deletions; likewise, the *rmpA* gene in some plasmids also possessed mutations. Surprisingly, most of the virulence plasmids do not encode a hypermucoviscous phenotype in these CRKP strains, presumably due to mutational changes in both virulence genes. However, some of the isolates which carried WT *rmpA* genes do not exhibit the hypermucoviscous phenotype, suggesting that specific genetic traits in either the virulence plasmid or chromosome of *K. pneumoniae* also play a role in regulating the expression of the hypermucoviscous and hypervirulence phenotypes of *K. pneumoniae*. Among the 30 phenotypic CR-HvKP strains sequenced, most carry a WT *rmpA* gene, whereas some carry various mutations of *rmpA2*, suggesting that a functional *rmpA* gene alone is sufficient for mediating expression of phenotypic hypervirulence. However, some phenotypic CR-HvKP strains only carry a mutated *rmpA2* gene, among which *rmpA2_M3*, *rmpA2_M5* and *rmpA2_M9* are known to contain deletion or insertion mutations, implying that mechanisms other than *rmpA/rmpA2* could also mediate hypermucoviscous phenotype (Table S2). Although *rmpA/rmpA2* were required for the expression of hypermucoviscosity in some *K. pneumoniae* strains, such as the ST86/K2 *K. pneumoniae* strain CG43, they were not a sufficient factor for this phenotype in some other genetic clones. It has been reported that mutations in genes involved in the regulation and biosynthesis of capsular polysaccharides could mediate the hypermucoviscosity phenotype, which might also contribute to hypervirulence [33,36]. However, we did not identify any strain with these mutations in this study. Further works are required to investigate the range of cellular mechanisms underlying the expression of hypervirulence in *K. pneumoniae*. In addition, consistent with previous reports, we identified three CRKP strains that carried virulence plasmid and exhibited the hypermucoviscous phenotype, but did not display high survival rate in neutrophil cells (<20%). We therefore do not define these three strains as phenotypic hypervirulence.

One limitation of this study is to screen for the strains by string test first and then selected the positive strains for neutrophil survival assay to determine their phenotypic hypervirulence due to the lack of other high throughput assays to identify phenotypic hypervirulence of CR-HvKP strains. The hypermucoviscosity phenotype has been identified as a virulence factor among clinical bacteremia isolates of *K. pneumoniae*. However, some of the CR-HvKP strains might not express hypermucoviscosity phenotype, these CR-HvKP strains would be screened out using this approach, which suggests that our data might under-estimate the prevalence of phenotypic CR-HvKP in these hospitals. Whereas screening by string test would enable us to pick up CR-HvKP strains that did not carry virulence plasmid. Our data suggest that positive detection of virulence plasmid or string test result do not necessarily reflect hypervirulence in *K. pneumoniae*. On the other hand, the absence of a virulence plasmid and negative string test results does not always indicate non-hypervirulence. The current genetic markers for HvKP identified in a report could not help correlating well in these batch of strains [37]. More reliable genetic markers of hypervirulence in *K. pneumoniae* need to be identified to facilitate rapid differentiation between hypervirulent and non-hypervirulent strains. This study also calls for a better definition and screening for these truly virulent CR-HvKP strains in the clinical setting.

## Supplementary Material

Supplemental Material

## Data Availability

Assembled genome sequences of the strains used in this study have been deposited in the GenBank database under BioProject number PRJNA503173.
