# Supplementary material for: Molecular epidemiology of carbapenem-resistant hypervirulent Klebsiella pneumoniae in China
Source: Emerg Microbes Infect. 2022 Mar 19;11(1):841–9. doi: 10.1080/22221751.2022.2049458 (PMC8942559; doi:10.1080/22221751.2022.2049458)
Supplement: Supplemental Material [file TEMI_A_2049458_SM3950.zip › Suppl files/CRHvKP Supplementary Materials_r1.docx]

Supplementary Materials

**Molecular epidemiology of carbapenem-resistant hypervirulent *Klebsiella pneumoniae* in China**

Xuemei Yang^1^, Qiaoling Sun^2,3^, Jiaping Li^2^, Yu Jiang^2^, Yi Li^4^, Jianping Lin^5^, Kaichao Chen^1^, Edward Wai-Chi Chan^6^, Rong Zhang^2*^ and Sheng Chen^1*^

# ^1^Department of Infectious Diseases and Public Health, City University of Hong Kong, Kowloon, Hong Kong;

# ^2^Department of Clinical Laboratory Medicine, Second Affiliated Hospital of Zhejiang University, Hangzhou, China;

# ^3^Department of Clinical Laboratory Medicine, Frist Affiliated Hospital of Zhejiang University, Hangzhou, China;

# ^4^Department of Clinical Laboratory Medicine, Henan Provincial People’s Hospital, Zhengzhou, China;

^5^Department of Clinical Laboratory Medicine, Wenzhou people’s Hospital, Wenzhou, Zhejiang, China

# ^6^State Key Laboratory of Chirosciences, Department of Applied Biology and Chemical Technology, The Hong Kong Polytechnic University, Hung Hom, Kowloon, Hong Kong;

# *Corresponding authors: Prof. Rong Zhang, [brigitte_zx@163.com](mailto:brigitte_zx@163.com), Department of Clinical Laboratory Medicine, Second Affiliated Hospital of Zhejiang University, Hangzhou, China; Prof. Sheng Chen, [shechen@cityu.edu.hk](mailto:shechen@cityu.edu.hk), Department of Infectious Diseases and Public Health, City University of Hong Kong, Kowloon, Hong Kong.

**Running title:** epidemiological study of CR-HvKP

**Keywords:** CR-HvKP, Prevalence, Epidemiology, Virulence plasmid

**Supplementary Table S1. Prevalence rate of carbapenem-resistant *Klebsiella pneumoniae* (CRKP) among all clinical *K. pneumoniae* (KP) strains recorded in three hospitals in China.**

| **Year** | **SAHZU** | | **HPPH** | | **WZTH** | |
| --- | --- | --- | --- | --- | --- | --- |
|  | **No of KP** | **No of CRKP (%)** | **No of KP** | **No of CRKP (%)** | **No of KP** | **No of CRKP (%)** |
| 2013 | 1380 | 276 (20.0) | 448 | 39 (8.7) | 658 | 40 (6.1) |
| 2014 | 1482 | 392 (26.5) | 524 | 82 (15.7) | 2366 | 94 (4.0) |
| 2015 | 1913 | 538 (28.1) | 591 | 212 (35.8) | 2896 | 230 (7.9) |
| 2016 | 1799 | 595 (33.1) | 813 | 389 (47.8) | 1746 | 166 (9.5) |
| 2017(1-7) | 844 | 306 (36.3) | 671 | 412 (61.4) | 2300 | 281 (12.2) |
| Total | 5619 | 2107 (37.5) | 3047 | 1134 (37.2) | 7208 | 811 (11.3) |

**
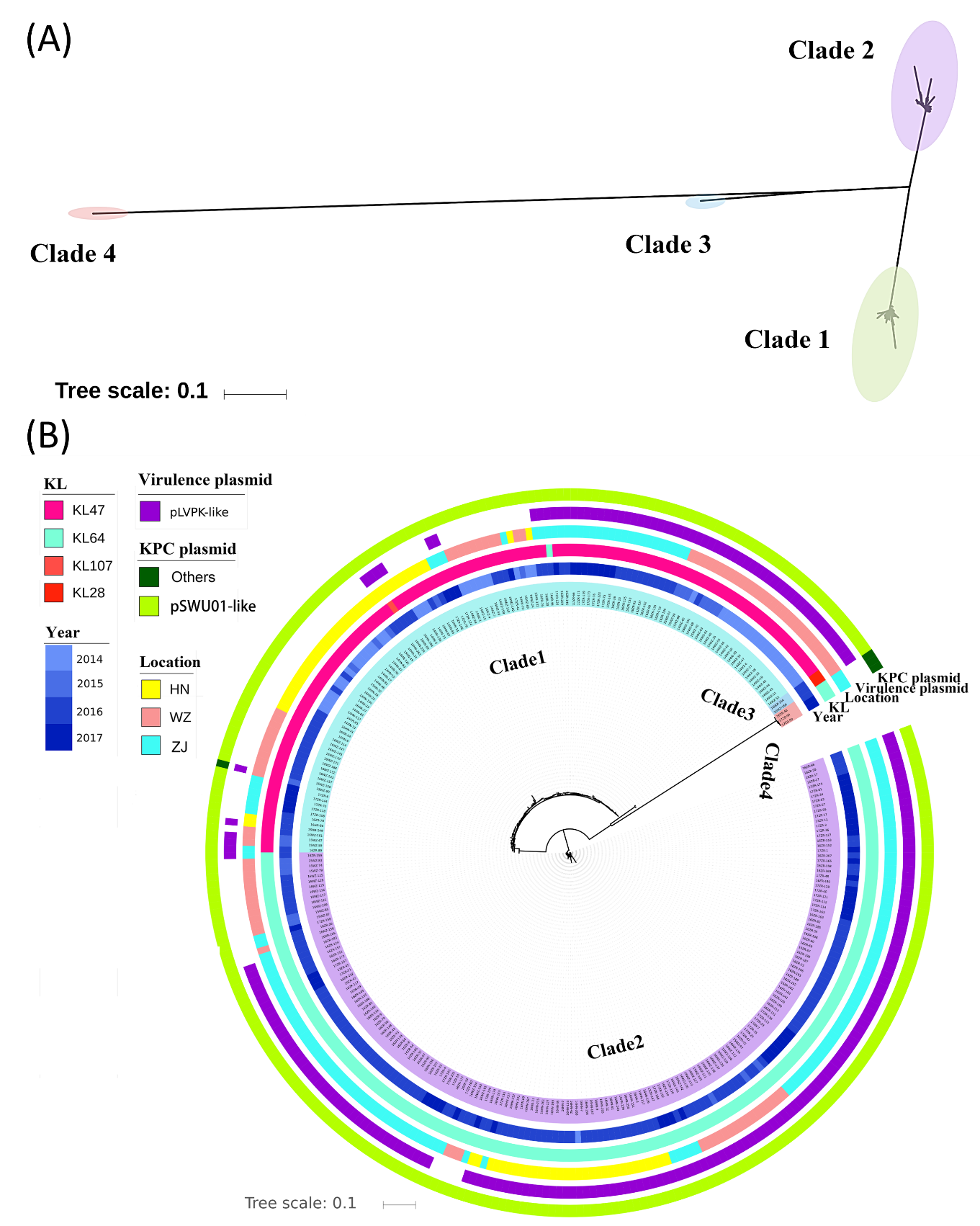
**

**Supplementary Figure S1. Genetic analysis of all ST11 CRKP strains.** (A) Four genetic clades were identified in ST11 CRKP strains. (B) Phylogenetic tree of ST11 CRKP strains recovered from three hospitals in China. The serotypes and location / year of isolation of the strains are shown.


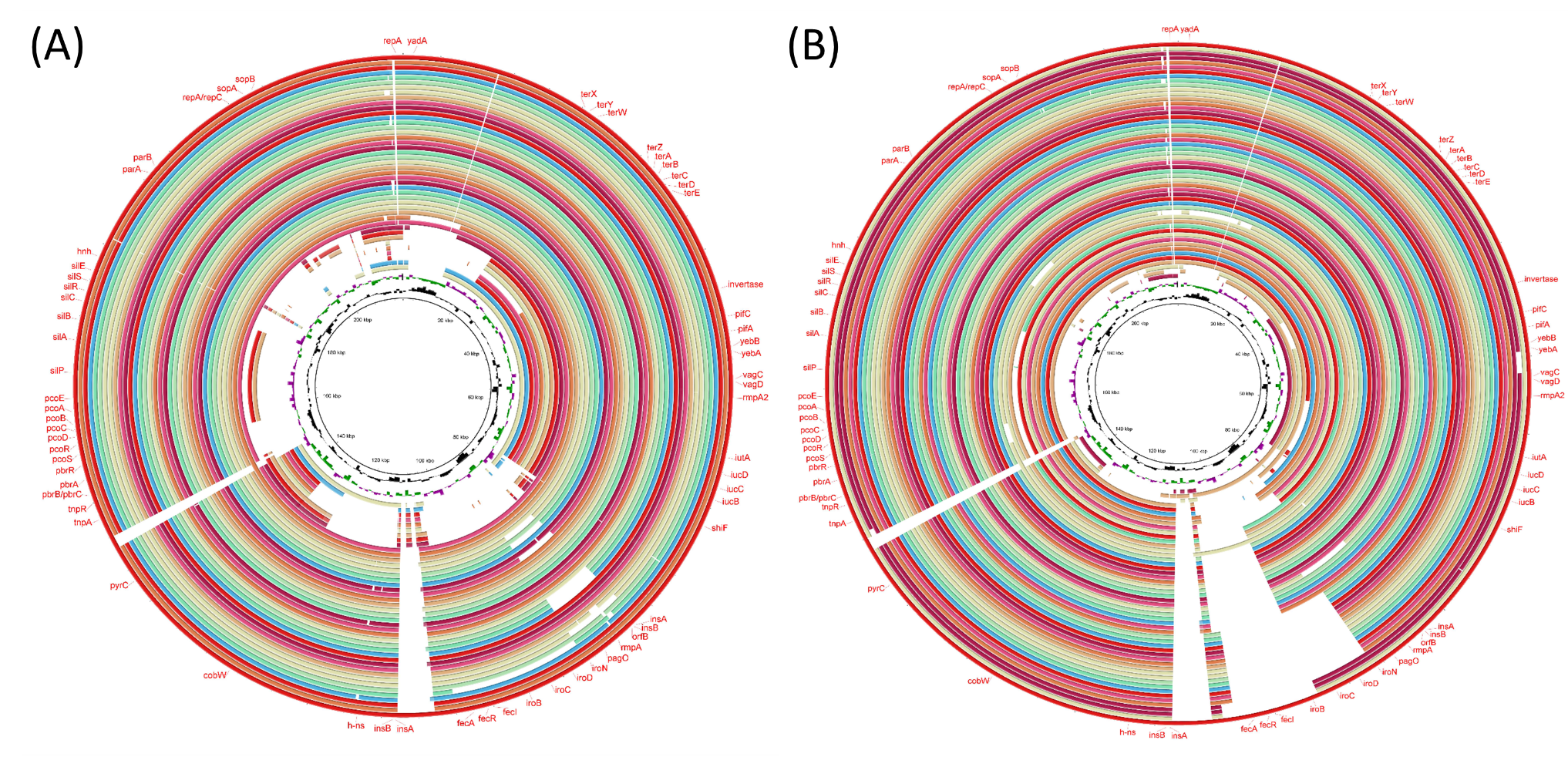


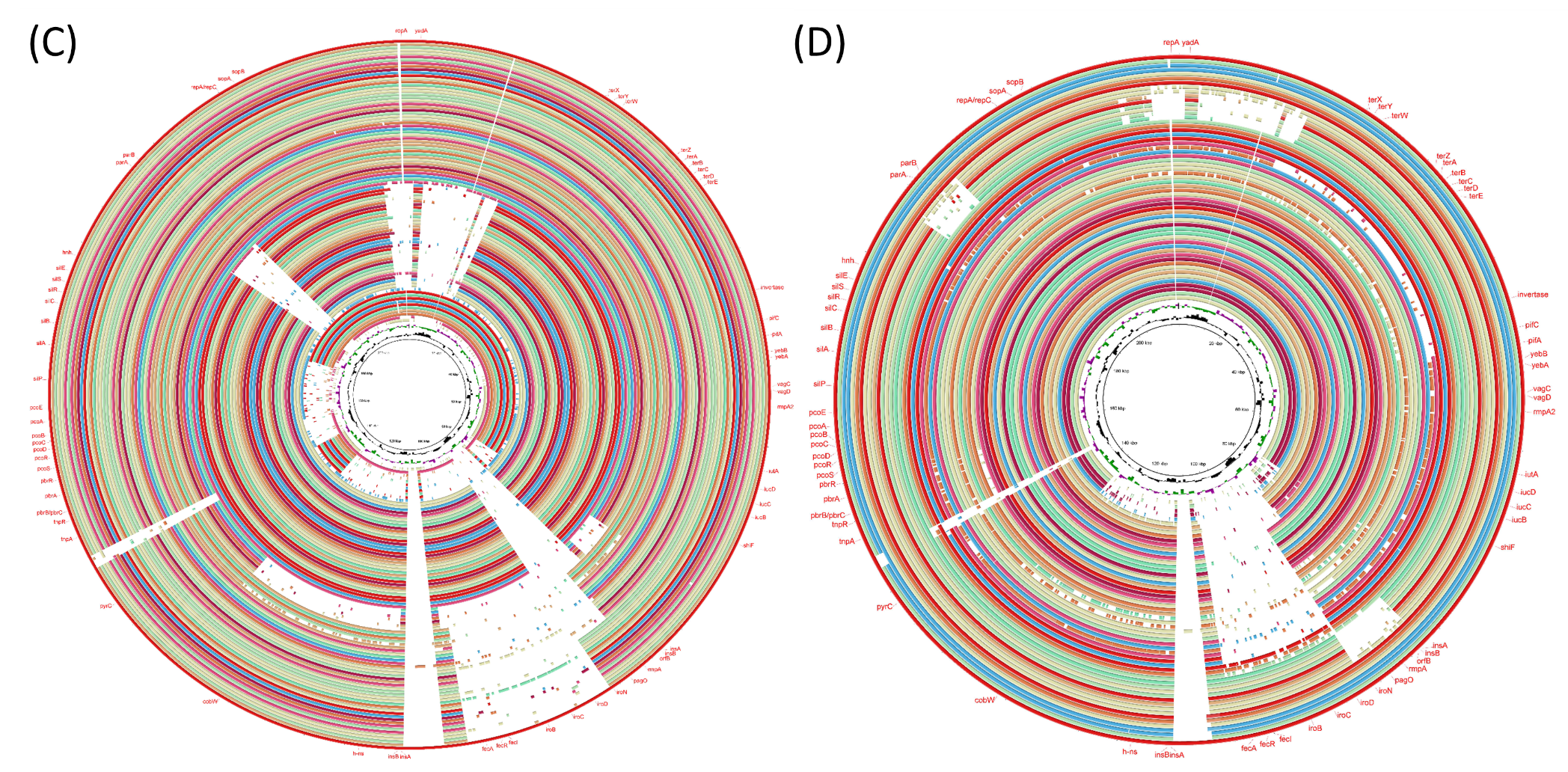
**Supplementary Figure S2. Alignment of virulence plasmids recovered from CRKP strains in three hospitals in China to the virulence plasmid pLVPK shows that virulence plasmids of CRKP strains in each hospital exhibited a unique genetic profile.** (A) Alignment of virulence plasmids from CRKP strains isolated in HPPH to the reference plasmid (pLVPK); (B) alignment of virulence plasmids from CRKP strains isolated in WZTH to the reference plasmid; alignment of virulence plasmids from CRKP strains isolated in SAHZU in 2016 (C) and 2017 (D) to the reference plasmid.

**(A) Summary of DNA mutations on *rmpA* and *rmpA2* genes**

| ***rmpA*** | **Substitutions** | ***rmpA2*** | **Substitutions** |
| --- | --- | --- | --- |
| WT |  | M1 | C^301^A mutation |
| M1 | G insertion at 286 | M2 | C^301^A, GGG deletion at 284-286 |
| M2 | G deletion at 285 | M3 | C^301^A, GGG deletion at 284-286 and G^358^A |
| M3 | N-terminal 94 bp deletion | M4 | C^301^A and A deletion at 353 |
| M4 | N-terminal 78 bp deletion | M5 | C^301^A, GG deletion at 285-286 and A deletion at 353 |
| M5 | C-terminal 67 bp deletion | M6 | C^301^A, GGG deletion at 284-286 and A deletion at 353 |
|  |  | M7 | C^301^A, A deletion at 353 and G^358^A |
|  |  | M8 | C^301^A and G insertion at 287 |
|  |  | M9 | C^301^A, GG insertion at 287 and G^204^A |

**(B) DNA sequence alignment for *rmpA* gene and its mutants**

rmpA ATGGAAAAATATATTTACTTTATATGTAACAAGGATGTAAACATAGTGTTGACTGATGAT 60

rmpA_M1 atggaaaaatatatttactttatatgtaacaaggatgtaaacatagtgttgactgatgat 60

rmpA_M2 atggaaaaatatatttactttatatgtaacaaggatgtaaacatagtgttgactgatgat 60

rmpA_M3 ------------------------------------------------------------ 0

rmpA_M4 ------------------------------------------------------------ 0

rmpA_M5 atggaaaaatatatttactttatatgtaacaaggatgtaaacatagtgttgactgatgat 60

rmpA TATTTTTTTTATTATGGCCTAAAGCAGTTAACTGGACTACCTCTGTTTCATATTACATAT 120

rmpA_M1 tattttttttattatggcctaaagcagttaactggactacctctgtttcatattacatat 120

rmpA_M2 tattttttttattatggcctaaagcagttaactggactacctctgtttcatattacatat 120

rmpA_M3 ----------------------------------gactacctctgtttcatattacatat 26

rmpA_M4 ------------------ctaaagcagttaactggactacctctgtttcatattacatat 42

rmpA_M5 tattttttttattatggcctaaagcagttaactggactacctctgtttcatattacatat 120

**************************

rmpA GAAGGAGTAGTTAATAAATCAATAGCAATTAAGCACAAAAGAAACATAAGAGTATTGGTT 180

rmpA_M1 gaaggagtagttaataaatcaatagcaattaagcacaaaagaaacataagagtattggtt 180

rmpA_M2 gaaggagtagttaataaatcaatagcaattaagcacaaaagaaacataagagtattggtt 180

rmpA_M3 gaaggagtagttaataaatcaatagcaattaagcacaaaagaaacataagagtattggtt 86

rmpA_M4 gaaggagtagttaataaatcaatagcaattaagcacaaaagaaacataagagtattggtt 102

rmpA_M5 gaaggagtagttaataaatcaatagcaattaagcacaaaagaaacataagagtattggtt 180

************************************************************

rmpA GACAGCAGGATTTTTTATTCAGGGAAATGGGGAGGGTACAAAATGTTAAGGGGATCATTA 240

rmpA_M1 gacagcaggattttttattcagggaaatggggagggtacaaaatgttaaggggatcatta 240

rmpA_M2 gacagcaggattttttattcagggaaatggggagggtacaaaatgttaaggggatcatta 240

rmpA_M3 gacagcaggattttttattcagggaaatggggagggtacaaaatgttaaggggatcatta 146

rmpA_M4 gacagcaggattttttattcagggaaatggggagggtacaaaatgttaaggggatcatta 162

rmpA_M5 gacagcaggattttttattcagggaaatggggagggtacaaaatgttaaggggatcatta 240

************************************************************

rmpA AATATGATAAGCCAATGGATGTGGCTTGACGTTTCGGGGGG-GGGGCGGTTTTATCCTAA 299

rmpA_M1 aatatgataagccaatggatgtggcttgacgtttcgggggggggggcggttttatcctaa 300

rmpA_M2 aatatgataagccaatggatgtggcttgacgtttcgggggg--gggcggttttatcctaa 298

rmpA_M3 aatatgataagccaatggatgtggcttgacgtttcgggggg-ggggcggttttatcctaa 205

rmpA_M4 aatatgataagccaatggatgtggcttgacgtttcgggggg-ggggcggttttatcctaa 221

rmpA_M5 aatatgataagccaatggatgtggcttgacgtttcgggggg-ggggcggttttatcctaa 299

***************************************** *****************

rmpA AGGGTGTGATTATGACATCTATGTTAACATGCAAGGAAATGTAAAAAATAATATTGAAAA 359

rmpA_M1 agggtgtgattatgacatctatgttaacatgcaaggaaatgtaaaaaataatattgaaaa 360

rmpA_M2 agggtgtgattatgacatctatgttaacatgcaaggaaatgtaaaaaataatattgaaaa 358

rmpA_M3 agggtgtgattatgacatctatgttaacatgcaaggaaatgtaaaaaataatattgaaaa 265

rmpA_M4 agggtgtgattatgacatctatgttaacatgcaaggaaatgtaaaaaataatattgaaaa 281

rmpA_M5 agggtgtgattatgacatctatgttaacatgcaaggaaatgtaaaaaataatattgaaaa 359

************************************************************

rmpA ACTATATTTTGCATTCTTAAAGAAAAATGTTAGCCGAATTGTAAACCATTATCCACGGCT 419

rmpA_M1 actatattttgcattcttaaagaaaaatgttagccgaattgtaaaccattatccacggct 420

rmpA_M2 actatattttgcattcttaaagaaaaatgttagccgaattgtaaaccattatccacggct 418

rmpA_M3 actatattttgcattcttaaagaaaaatgttagccgaattgtaaaccattatccacggct 325

rmpA_M4 actatattttgcattcttaaagaaaaatgttagccgaattgtaaaccattatccacggct 341

rmpA_M5 actatattttgcattcttaaagaaaaatgttagccgaattgtaaaccattatccacggct 419

************************************************************

rmpA AACAAAAAAGGAACAAGCAGTGCTGCAATGCCTACTGAAAAATGGGGGCATTAATGAAAT 479

rmpA_M1 aacaaaaaaggaacaagcagtgctgcaatgcctactgaaaaatgggggcattaatgaaat 480

rmpA_M2 aacaaaaaaggaacaagcagtgctgcaatgcctactgaaaaatgggggcattaatgaaat 478

rmpA_M3 aacaaaaaaggaacaagcagtgctgcaatgcctactgaaaaatgggggcattaatgaaat 385

rmpA_M4 aacaaaaaaggaacaagcagtgctgcaatgcctactgaaaaatgggggcattaatgaaat 401

rmpA_M5 aacaaaaaaggaacaagcagtgctgcaatgcctactgaaaaatgggggcattaatgaaat 479

************************************************************

rmpA AAAAAGTCAACTAAAAATTGAAGAAAAAACGCTATCATGCTACCAAAGCAAAATAACAAG 539

rmpA_M1 aaaaagtcaactaaaaattgaagaaaaaacgctatcatgctaccaaagcaaaataacaag 540

rmpA_M2 aaaaagtcaactaaaaattgaagaaaaaacgctatcatgctaccaaagcaaaataacaag 538

rmpA_M3 aaaaagtcaactaaaaattgaagaaaaaacgctatcatgctaccaaagcaaaataacaag 445

rmpA_M4 aaaaagtcaactaaaaattgaagaaaaaacgctatcatgctaccaaagcaaaataacaag 461

rmpA_M5 aaaaagtcaactaaaaattgaagaaaaaacgctatcatgctaccaaagcaaaataacaag 539

************************************************************

rmpA AAAATTTGGCTGCAAAAGGTACATAAGGTTTATGTATCTTTACAGCCTTAATAAAGAAAT 599

rmpA_M1 aaaatttggctgcaaaaggtacataaggtttatgtatctttacagccttaataaagaaat 600

rmpA_M2 aaaatttggctgcaaaaggtacataaggtttatgtatctttacagccttaataaagaaat 598

rmpA_M3 aaaatttggctgcaaaaggtacataaggtttatgtatctttacagccttaataaagaaat 505

rmpA_M4 aaaatttggctgcaaaaggtacataaggtttatgtatctttacagccttaataaagaaat 521

rmpA_M5 aaaatttggctgcaaaaggtacataagg-------------------------------- 566

****************************

rmpA GGTTGATGAAAGATGGCTCATGCCAAGTATTTAG 633

rmpA_M1 ggttgatgaaagatggctcatgccaagtatttag 634

rmpA_M2 ggttgatgaaagatggctcatgccaagtatttag 632

rmpA_M3 ggttgatgaaagatggctcatgccaagtatttag 539

rmpA_M4 ggttgatgaaagatggctcatgccaagtatttag 555

rmpA_M5 ---------------------------------- 566

**(C) DNA sequence alignment of *rmpA2* gene and its mutants**

rmpA2 ATGGAAAAATATATTTACTTTATGTGCAATAAGGATGTTACATTAGTGTTAACTGATGAT 60

rmpA2_M1 atggaaaaatatatttactttatgtgcaataaggatgttacattagtgttaactgatgat 60

rmpA2_M2 atggaaaaatatatttactttatgtgcaataaggatgttacattagtgttaactgatgat 60

rmpA2_M3 atggaaaaatatatttactttatgtgcaataaggatgttacattagtgttaactgatgat 60

rmpA2_M4 atggaaaaatatatttactttatgtgcaataaggatgttacattagtgttaactgatgat 60

rmpA2_M5 atggaaaaatatatttactttatgtgcaataaggatgttacattagtgttaactgatgat 60

rmpA2_M6 atggaaaaatatatttactttatgtgcaataaggatgttacattagtgttaactgatgat 60

rmpA2_M7 atggaaaaatatatttactttatgtgcaataaggatgttacattagtgttaactgatgat 60

rmpA2_M8 atggaaaaatatatttactttatgtgcaataaggatgttacattagtgttaactgatgat 60

rmpA2_M9 atggaaaaatatatttactttatgtgcaataaggatgttacattagtgttaactgatgat 60

************************************************************

rmpA2 TATTATTTTTATTTCGGCCTAAAGCAGTTAACTGGACTACCTCTGGTTTATATTACGTAT 120

rmpA2_M1 tattatttttatttcggcctaaagcagttaactggactacctctggtttatattacgtat 120

rmpA2_M2 tattatttttatttcggcctaaagcagttaactggactacctctggtttatattacgtat 120

rmpA2_M3 tattatttttatttcggcctaaagcagttaactggactacctctggtttatattacgtat 120

rmpA2_M4 tattatttttatttcggcctaaagcagttaactggactacctctggtttatattacgtat 120

rmpA2_M5 tattatttttatttcggcctaaagcagttaactggactacctctggtttatattacgtat 120

rmpA2_M6 tattatttttatttcggcctaaagcagttaactggactacctctggtttatattacgtat 120

rmpA2_M7 tattatttttatttcggcctaaagcagttaactggactacctctggtttatattacgtat 120

rmpA2_M8 tattatttttatttcggcctaaagcagttaactggactacctctggtttatattacgtat 120

rmpA2_M9 tattatttttatttcggcctaaagcagttaactggactacctctggtttatattacgtat 120

************************************************************

rmpA2 GAAGGCTCGATGGATAAACCAATAGTTATTAAGCAGAAAAGAAATATAAGAGTATTGGTT 180

rmpA2_M1 gaaggctcgatggataaaccaatagttattaagcagaaaagaaatataagagtattggtt 180

rmpA2_M2 gaaggctcgatggataaaccaatagttattaagcagaaaagaaatataagagtattggtt 180

rmpA2_M3 gaaggctcgatggataaaccaatagttattaagcagaaaagaaatataagagtattggtt 180

rmpA2_M4 gaaggctcgatggataaaccaatagttattaagcagaaaagaaatataagagtattggtt 180

rmpA2_M5 gaaggctcgatggataaaccaatagttattaagcagaaaagaaatataagagtattggtt 180

rmpA2_M6 gaaggctcgatggataaaccaatagttattaagcagaaaagaaatataagagtattggtt 180

rmpA2_M7 gaaggctcgatggataaaccaatagttattaagcagaaaagaaatataagagtattggtt 180

rmpA2_M8 gaaggctcgatggataaaccaatagttattaagcagaaaagaaatataagagtattggtt 180

rmpA2_M9 gaaggctcgatggataaaccaatagttattaagcagaaaagaaatataagagtattggtt 180

************************************************************

rmpA2 GATAGCCGGATTTTTTATTCAGGGAAATGGGATGGTTATAAAATGTTAAGGAAAACATTA 240

rmpA2_M1 gatagccggattttttattcagggaaatgggatggttataaaatgttaaggaaaacatta 240

rmpA2_M2 gatagccggattttttattcagggaaatgggatggttataaaatgttaaggaaaacatta 240

rmpA2_M3 gatagccggattttttattcagggaaatgggatggttataaaatgttaaggaaaacatta 240

rmpA2_M4 gatagccggattttttattcagggaaatgggatggttataaaatgttaaggaaaacatta 240

rmpA2_M5 gatagccggattttttattcagggaaatgggatggttataaaatgttaaggaaaacatta 240

rmpA2_M6 gatagccggattttttattcagggaaatgggatggttataaaatgttaaggaaaacatta 240

rmpA2_M7 gatagccggattttttattcagggaaatgggatggttataaaatgttaaggaaaacatta 240

rmpA2_M8 gatagccggattttttattcagggaaatgggatggttataaaatgttaaggaaaacatta 240

rmpA2_M9 gatagccggattttttattcaggaaaatgggatggttataaaatgttaaggaaaacatta 240

*********************** ************************************

rmpA2 AATATGATAAGTCAATGGATGTGGCTTGACATTTCGGGGGGGGGGG--AGAAGTTTTATC 298

rmpA2_M1 aatatgataagtcaatggatgtggcttgacatttcggggggggggg--agaagttttatc 298

rmpA2_M2 aatatgataagtcaatggatgtggcttgacatttcgggggggg-----agaagttttatc 295

rmpA2_M3 aatatgataagtcaatggatgtggcttgacatttcgggggggg-----agaagttttatc 295

rmpA2_M4 aatatgataagtcaatggatgtggcttgacatttcggggggggggg--agaagttttatc 298

rmpA2_M5 aatatgataagtcaatggatgtggcttgacatttcggggggggg----agaagttttatc 296

rmpA2_M6 aatatgataagtcaatggatgtggcttgacatttcgggggggg-----agaagttttatc 295

rmpA2_M7 aatatgataagtcaatggatgtggcttgacatttcggggggggggg--agaagttttatc 298

rmpA2_M8 aatatgataagtcaatggatgtggcttgacatttcgggggggggggg-agaagttttatc 299

rmpA2_M9 aatatgataagtcaatggatgtggcttgacatttcgggggggggggggagaagttttatc 300

******************************************* ************

rmpA2 CTCAAGGGTGTGATTATGACATCTATGTCAACATGCAAGGAAATTTAAAAAAAAACATTG 358

rmpA2_M1 ctaaagggtgtgattatgacatctatgtcaacatgcaaggaaatttaaaaaaaaacattg 358

rmpA2_M2 ctaaagggtgtgattatgacatctatgtcaacatgcaaggaaatttaaaaaaaaacattg 355

rmpA2_M3 ctaaagggtgtgattatgacatctatgtcaacatgcaaggaaatttaaaaaaaaacatta 355

rmpA2_M4 ctaaagggtgtgattatgacatctatgtcaacatgcaaggaaatttaaaaaaaa-cattg 357

rmpA2_M5 ctaaagggtgtgattatgacatctatgtcaacatgcaaggaaatttaaaaaaaa-cattg 355

rmpA2_M6 ctaaagggtgtgattatgacatctatgtcaacatgcaaggaaatttaaaaaaaa-cattg 354

rmpA2_M7 ctaaagggtgtgattatgacatctatgtcaacatgcaaggaaatttaaaaaaaa-catta 357

rmpA2_M8 ctaaagggtgtgattatgacatctatgtcaacatgcaaggaaatttaaaaaaaaacattg 359

rmpA2_M9 ctaaagggtgtgattatgacatctatgtcaacatgcaaggaaatttaaaaaaaaacattg 360

** *************************************************** ****

rmpA2 AAGAGCTATATTATGCATACTTAAAGAAAAATGTTAGCCGGATTGGAAATCATTACCCAC 418

rmpA2_M1 aagagctatattatgcatacttaaagaaaaatgttagccggattggaaatcattacccac 418

rmpA2_M2 aagagctatattatgcatacttaaagaaaaatgttagccggattggaaatcattacccac 415

rmpA2_M3 aagagctatattatgcatacttaaagaaaaatgttagccggattggaaatcattacccac 415

rmpA2_M4 aagagctatattatgcatacttaaagaaaaatgttagccggattggaaatcattacccac 417

rmpA2_M5 aagagctatattatgcatacttaaagaaaaatgttagccggattggaaatcattacccac 415

rmpA2_M6 aagagctatattatgcatacttaaagaaaaatgttagccggattggaaatcattacccac 414

rmpA2_M7 aagagctatattatgcatacttaaagaaaaatgttagccggattggaaatcattacccac 417

rmpA2_M8 aagagctatattatgcatacttaaagaaaaatgttagccggattggaaatcattacccac 419

rmpA2_M9 aagagctatattatgcatacttaaagaaaaatgttagccggattggaaatcattacccac 420

************************************************************

rmpA2 AACTAACAAAAAAAGAACAAATCATTCTACAATGCTTACTCTCCAGGAGGGAGGGCATCC 478

rmpA2_M1 aactaacaaaaaaagaacaaatcattctacaatgcttactctccaggagggagggcatcc 478

rmpA2_M2 aactaacaaaaaaagaacaaatcattctacaatgcttactctccaggagggagggcatcc 475

rmpA2_M3 aactaacaaaaaaagaacaaatcattctacaatgcttactctccaggagggagggcatcc 475

rmpA2_M4 aactaacaaaaaaagaacaaatcattctacaatgcttactctccaggagggagggcatcc 477

rmpA2_M5 aactaacaaaaaaagaacaaatcattctacaatgcttactctccaggagggagggcatcc 475

rmpA2_M6 aactaacaaaaaaagaacaaatcattctacaatgcttactctccaggagggagggcatcc 474

rmpA2_M7 aactaacaaaaaaagaacaaatcattctacaatgcttactctccaggagggagggcatcc 477

rmpA2_M8 aactaacaaaaaaagaacaaatcattctacaatgcttactctccaggagggagggcatcc 479

rmpA2_M9 aactaacaaaaaaagaacaaatcattctacaatgcttactctccaggagggagggcatcc 480

************************************************************

rmpA2 ATGAATTAAAAAGCCGTCTAAAAATTGAAGAGAAAACACTATCGTGTCACAGATGTAAAA 538

rmpA2_M1 atgaattaaaaagccgtctaaaaattgaagagaaaacactatcgtgtcacagatgtaaaa 538

rmpA2_M2 atgaattaaaaagccgtctaaaaattgaagagaaaacactatcgtgtcacagatgtaaaa 535

rmpA2_M3 atgaattaaaaagccgtctaaaaattgaagagaaaacactatcgtgtcacagatgtaaaa 535

rmpA2_M4 atgaattaaaaagccgtctaaaaattgaagagaaaacactatcgtgtcacagatgtaaaa 537

rmpA2_M5 atgaattaaaaagccgtctaaaaattgaagagaaaacactatcgtgtcacagatgtaaaa 535

rmpA2_M6 atgaattaaaaagccgtctaaaaattgaagagaaaacactatcgtgtcacagatgtaaaa 534

rmpA2_M7 atgaattaaaaagccgtctaaaaattgaagagaaaacactatcgtgtcacagatgtaaaa 537

rmpA2_M8 atgaattaaaaagccgtctaaaaattgaagagaaaacactatcgtgtcacagatgtaaaa 539

rmpA2_M10 atgaattaaaaagccgtctaaaaattgaagagaaaacactatcgtgtcacagatgtaaaa 540

************************************************************

rmpA2 TAACAAGAAAATTTGGTTGTAAAAGATTCATAAGATTTATGTATCTTTACAACTTAAATA 598

rmpA2_M1 taacaagaaaatttggttgtaaaagattcataagatttatgtatctttacaacttaaata 598

rmpA2_M2 taacaagaaaatttggttgtaaaagattcataagatttatgtatctttacaacttaaata 595

rmpA2_M3 taacaagaaaatttggttgtaaaagattcataagatttatgtatctttacaacttaaata 595

rmpA2_M4 taacaagaaaatttggttgtaaaagattcataagatttatgtatctttacaacttaaata 597

rmpA2_M5 taacaagaaaatttggttgtaaaagattcataagatttatgtatctttacaacttaaata 595

rmpA2_M6 taacaagaaaatttggttgtaaaagattcataagatttatgtatctttacaacttaaata 594

rmpA2_M7 taacaagaaaatttggttgtaaaagattcataagatttatgtatctttacaacttaaata 597

rmpA2_M8 taacaagaaaatttggttgtaaaagattcataagatttatgtatctttacaacttaaata 599

rmpA2_M9 taacaagaaaatttggttgtaaaagattcataagatttatgtatctttacaacttaaata 560

************************************************************

rmpA2 AAGAAATAACTGATGAAAAATGGTGCACATCAAATACCTAG 639

rmpA2_M1 aagaaataactgatgaaaaatggtgcacatcaaatacctag 639

rmpA2_M2 aagaaataactgatgaaaaatggtgcacatcaaatacctag 636

rmpA2_M3 aagaaataactgatgaaaaatggtgcacatcaaatacctag 636

rmpA2_M4 aagaaataactgatgaaaaatggtgcacatcaaatacctag 638

rmpA2_M5 aagaaataactgatgaaaaatggtgcacatcaaatacctag 636

rmpA2_M6 aagaaataactgatgaaaaatggtgcacatcaaatacctag 635

rmpA2_M7 aagaaataactgatgaaaaatggtgcacatcaaatacctag 638

rmpA2_M8 aagaaataactgatgaaaaatggtgcacatcaaatacctag 640

rmpA2_M9 aagaaataactgatgaaaaatggtgcacatcaaatacctag 640

*****************************************

**(D) Amino acid sequence alignment of RmpA and its mutants**

rmpA MEKYIYFICNKDVNIVLTDDYFFYYGLKQLTGLPLFHITYEGVVNKSIAIKHKRNIRVLV

rmpA_M1 MEKYIYFICNKDVNIVLTDDYFFYYGLKQLTGLPLFHITYEGVVNKSIAIKHKRNIRVLV

rmpA_M2 MEKYIYFICNKDVNIVLTDDYFFYYGLKQLTGLPLFHITYEGVVNKSIAIKHKRNIRVLV

rmpA_M3 -----------------------------HHRLPLFHITYEGVVNKSIAIKHKRNIRVLV

rmpA_M4 ----------------SHIRNLFAPSLKQLTGLPLFHITYEGVVNKSIAIKHKRNIRVLV

rmpA_M5 MEKYIYFICNKDVNIVLTDDYFFYYGLKQLTGLPLFHITYEGVVNKSIAIKHKRNIRVLV

****************************

rmpA DSRIFYSGKWGGYKMLRGSLNMISQWMWLDVSGGGRFYPKGCDYDIYVNMQGNVKNNIEK

rmpA_M1 DSRIFYSGKWGGYKMLRGSLNMISQWMWLDVSGGGAVLS-RV-L-HLC-HARKCKK-Y-K

rmpA_M2 DSRIFYSGKWGGYKMLRGSLNMISQWMWLDVSGGGGFILKGVIMTSMLTCKEM-KIILKN

rmpA_M3 DSRIFYSGKWGGYKMLRGSLNMISQWMWLDVSGGGRFYPKGCDYDIYVNMQGNVKNNIEK

rmpA_M4 DSRIFYSGKWGGYKMLRGSLNMISQWMWLDVSGGGRFYPKGCDYDIYVNMQGNVKNNIEK

rmpA_M5 DSRIFYSGKWGGYKMLRGSLNMISQWMWLDVSGGGRFYPKGCDYDIYVNMQGNVKNNIEK

*********************************** . * :

rmpA LYFAFLKKNVSRIVNHYPRLTKKEQAVL-QCLLKNGGINEIKSQLKIEEKTLSCYQSKIT

rmpA_M1 TIFCILKEKC-PNCKPLSTANKKGTSSAAMPTEKWGH--NKKS----------------T

rmpA_M2 YILHS-RKMLAEL-TIIHG-QKRNKQCC-NAY-KMGALMK-KVN-KLKKKRYHATKAK-Q

rmpA_M3 LYFAFLKKNVSRIVNHYPRLTKKEQAVL-QCLLKNGGINEIKSQLKIEEKTLSCYQSKIT

rmpA_M4 LYFAFLKKNVSRIVNHYPRLTKKEQAVL-QCLLKNGGINEIKSQLKIEEKTLSCYQSKIT

rmpA_M5 LYFAFLKKNVSRIVNHYPRLTKKEQAVL-QCLLKNGGINEIKSQLKIEEKTLSCYQSKIT

: :: . *: * * : *

rmpA RKFGCKRYIRFMYLYSLNKEMVDERWLMPSI----------------------- 210

rmpA_M1 KN--RKN--AI----MLPKQNNKKIWLQKVHKVYVSLQP--RNG--KMAHAKYL 197

rmpA_M2 ENLAAKGT-GLCIF-----TALIKKWLMKDGSCQVF------------------ 201

rmpA_M3 RKFGCKRYIRFMYLYSLNKEMVDERWLMPSI----------------------- 181

rmpA_M4 RKFGCKRYIRFMYLYSLNKEMVDERWLMPSI----------------------- 194

rmpA_M5 RKFGCKRYIRLVMLPTY------------------------------------- 196

.: * : ** **

**(E) Amino acid sequence alignment of RmpA2 and its mutants**

rmpA2 MEKYIYFMCNKDVTLVLTDDYYFYFGLKQLTGLPLVYITYEGSMDKPIVIKQKRNIRVLV

rmpA2_M1 MEKYIYFMCNKDVTLVLTDDYYFYFGLKQLTGLPLVYITYEGSMDKPIVIKQKRNIRVLV

rmpA2_M2 MEKYIYFMCNKDVTLVLTDDYYFYFGLKQLTGLPLVYITYEGSMDKPIVIKQKRNIRVLV

rmpA2_M3 MEKYIYFMCNKDVTLVLTDDYYFYFGLKQLTGLPLVYITYEGSMDKPIVIKQKRNIRVLV

rmpA2_M4 MEKYIYFMCNKDVTLVLTDDYYFYFGLKQLTGLPLVYITYEGSMDKPIVIKQKRNIRVLV

rmpA2_M5 MEKYIYFMCNKDVTLVLTDDYYFYFGLKQLTGLPLVYITYEGSMDKPIVIKQKRNIRVLV

rmpA2_M6 MEKYIYFMCNKDVTLVLTDDYYFYFGLKQLTGLPLVYITYEGSMDKPIVIKQKRNIRVLV

rmpA2_M7 MEKYIYFMCNKDVTLVLTDDYYFYFGLKQLTGLPLVYITYEGSMDKPIVIKQKRNIRVLV

rmpA2_M8 MEKYIYFMCNKDVTLVLTDDYYFYFGLKQLTGLPLVYITYEGSMDKPIVIKQKRNIRVLV

rmpA2_M9 MEKYIYFMCNKDVTLVLTDDYYFYFGLKQLTGLPLVYITYEGSMDKPIVIKQKRNIRVLV

************************************************************

rmpA2 DSRIFYSGKWDGYKMLRKTLNMISQWMWLDISGGGEK-FYPQGCDYDIYVNMQGNLKKNI

rmpA2_M1 DSRIFYSGKWDGYKMLRKTLNMISQWMWLDISGGGEK-FYPKGCDYDIYVNMQGNLKKNI

rmpA2_M2 DSRIFYSGKWDGYKMLRKTLNMISQWMWLDISG-GEK-FYPKGCDYDIYVNMQGNLKKNI

rmpA2_M3 DSRIFYSGKWDGYKMLRKTLNMISQWMWLDISG-GEK-FYPKGCDYDIYVNMQGNLKKNI

rmpA2_M4 DSRIFYSGKWDGYKMLRKTLNMISQWMWLDISGGGEK-FYPKGCDYDIYVNMQGNLKKTL

rmpA2_M5 DSRIFYSGKWDGYKMLRKTLNMISQWMWLDISGGGEV-LS-RV--L-HLCQHARKFKKNI

rmpA2_M6 DSRIFYSGKWDGYKMLRKTLNMISQWMWLDISG-GEK-FYPKGCDYDIYVNMQGNLKKTL

rmpA2_M7 DSRIFYSGKWDGYKMLRKTLNMISQWMWLDISGGGEK-FYPKGCDYDIYVNMQGNLKKTL

rmpA2_M8 DSRIFYSGKWDGYKMLRKTLNMISQWMWLDISGGGGE-VLS-RV-L-HLCQHARKFKKKH

rmpA2_M9 DSRIFYSGKWDGYKMLRKTLNMISQWMWLDISGGGGRSFILKGVIMTSMSTCKEI-KKTL

********************************* * . **.

rmpA2 EELYYAYLKKNVSRIGNHYPQLTKKE-QIILQC--LLSRREG-IHELKSR-LK-IEEKTL

rmpA2_M1 EELYYAYLKKNVSRIGNHYPQLTKKE-QIILQC--LLSRREG-IHELKSR-LK-IEEKTL

rmpA2_M2 EELYYAYLKKNVSRIGNHYPQLTKKE-QIILQC--LLSRREG-IHELKSR-LK-IEEKTL

rmpA2_M3 KELYYAYLKKNVSRIGNHYPQLTKKE-QIILQC--LLSRREG-IHELKSR-LK-IEEKTL

rmpA2_M4 KSYIMHT-RKMLAGLE----IITHN-QKK--NKSFYNAYSPGGRASMN-KAV-KLKRKHY

rmpA2_M5 EELYYAYLKKNVSRIGNHYPQLTKKE-QIILQC--LLSRREG-IHELKSR-LK-IEEKTL

rmpA2_M6 KSYIMHT-RKMLAGLE----IITHN-QKK--NKSFYNAYSPGGRASMN-KAV-KLKRKHY

rmpA2_M7 NRYIIHT-RKMEKYIY----FMCNKDVTLVLTDDYYFYFGLKQLTGLP---LV------Y

rmpA2_M8 -RAILCILKEKC-PDWKSLPTTNKKR-TN-HST--MLTLQEG-GHP-IKKPSKN-RENTI

rmpA2_M9 KSYIMHT-RKMLAGLE----IITHN-QKK--NKSFYNAYSPGGRASMN-KAV-KLKRKHY

:: ::

rmpA2 -SCHRC--------K---------ITRKFGCKRFIRFMYLYNLNKEITDEKWCTSNT---

rmpA2_M1 -SCHRC--------K---------ITRKFGCKRFIRFMYLYNLNKEITDEKWCTSNT---

rmpA2_M2 -SCHRC--------K---------ITRKFGCKRFIRFMYLYNLNKEITDEKWCTSNT---

rmpA2_M3 -SCHRC--------K---------ITRKFGCKRFIRFMYLYNLNKEITDEKWCTSNT---

rmpA2_M4 RVTDVK-QENLVVKDS-DLC-------IFTT---IKK-LMKNGAHQIPRWKNIFT-----

rmpA2_M5 -SCHRC--------K---------ITRKFGCKRFIRFMYLYNLNKEITDEKWCTSNT-ME

rmpA2_M6 RVTDVK-QENLVVKDS-DLC-------IFTT---IKK-LMKNGAHQIPRWKNIFT-----

rmpA2_M7 ITYEGSMDKPIVIKQKRNIRV-LVDSRIFYSGKWDGYKMLRKTLNMISQWMWLD------

rmpA2_M8 -VSQM-NNKKIWL-KIHKIYVSLQLK-RNN--KMVHIKYL--------------------

rmpA2_M9 RVTDVK-QENLVVKDS-DLC-------IFTT---IKK-LMKNGAHQIP------------

. . : *

rmpA2 ----- 212

rmpA2_M1 ----- 212

rmpA2_M2 ----- 211

rmpA2_M3 ----- 211

rmpA2_M4 ----- 211

rmpA2_M5 KYIYF 215

rmpA2_M6 ----- 210

rmpA2_M7 ----- 218

rmpA2_M8 ----- 201

rmpA2_M9 ----- 204

**Supplementary Figure S3. DNA and amino acid sequence alignment of *rmpA*, *rmpA2* and their mutants.**


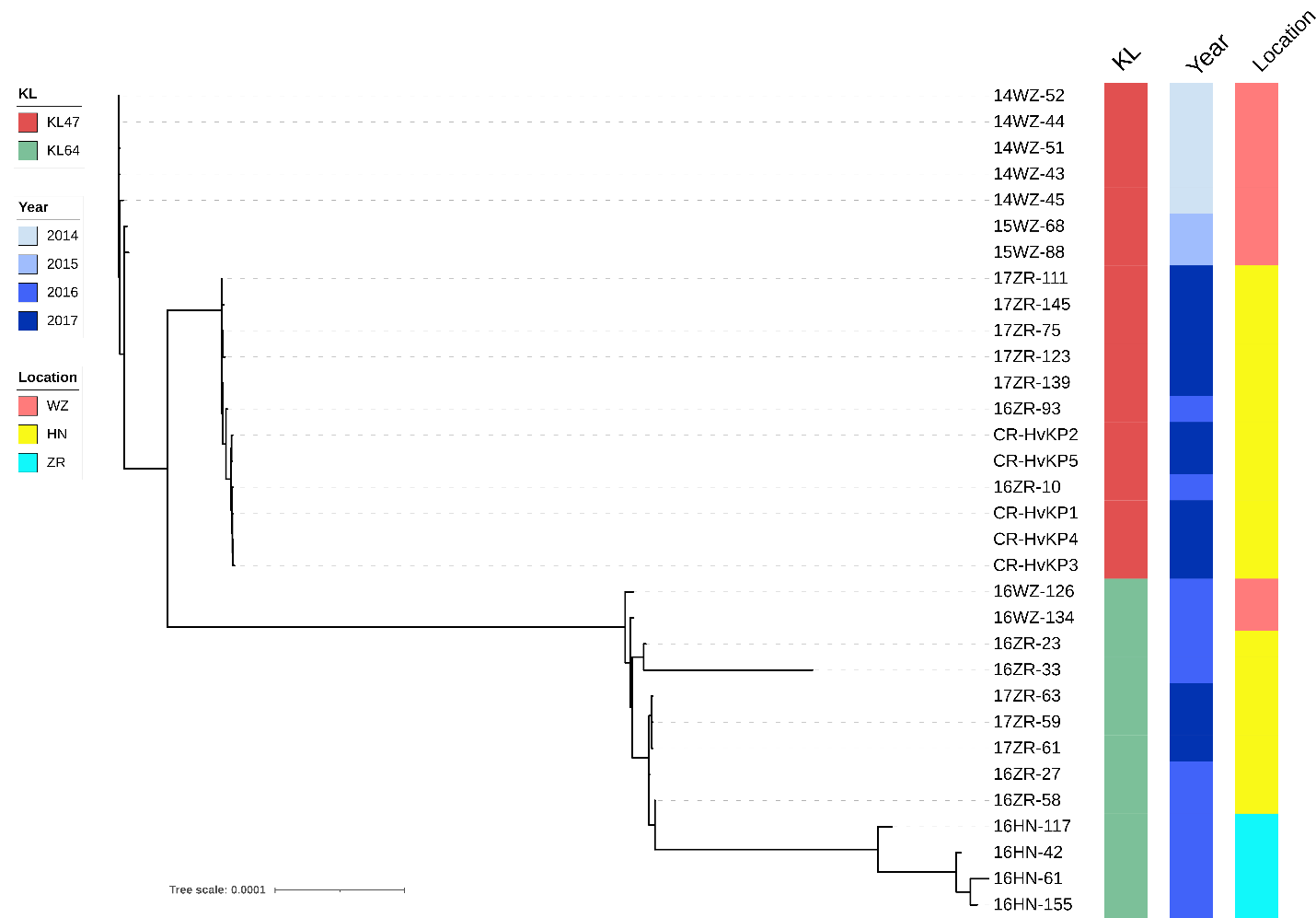


**Supplementary Figure S4.** Phylogenetic tree of ST11 phenotypic CR-HvKP strains recovered from three hospitals in China. The serotypes and location / year of isolation of the strains are shown.


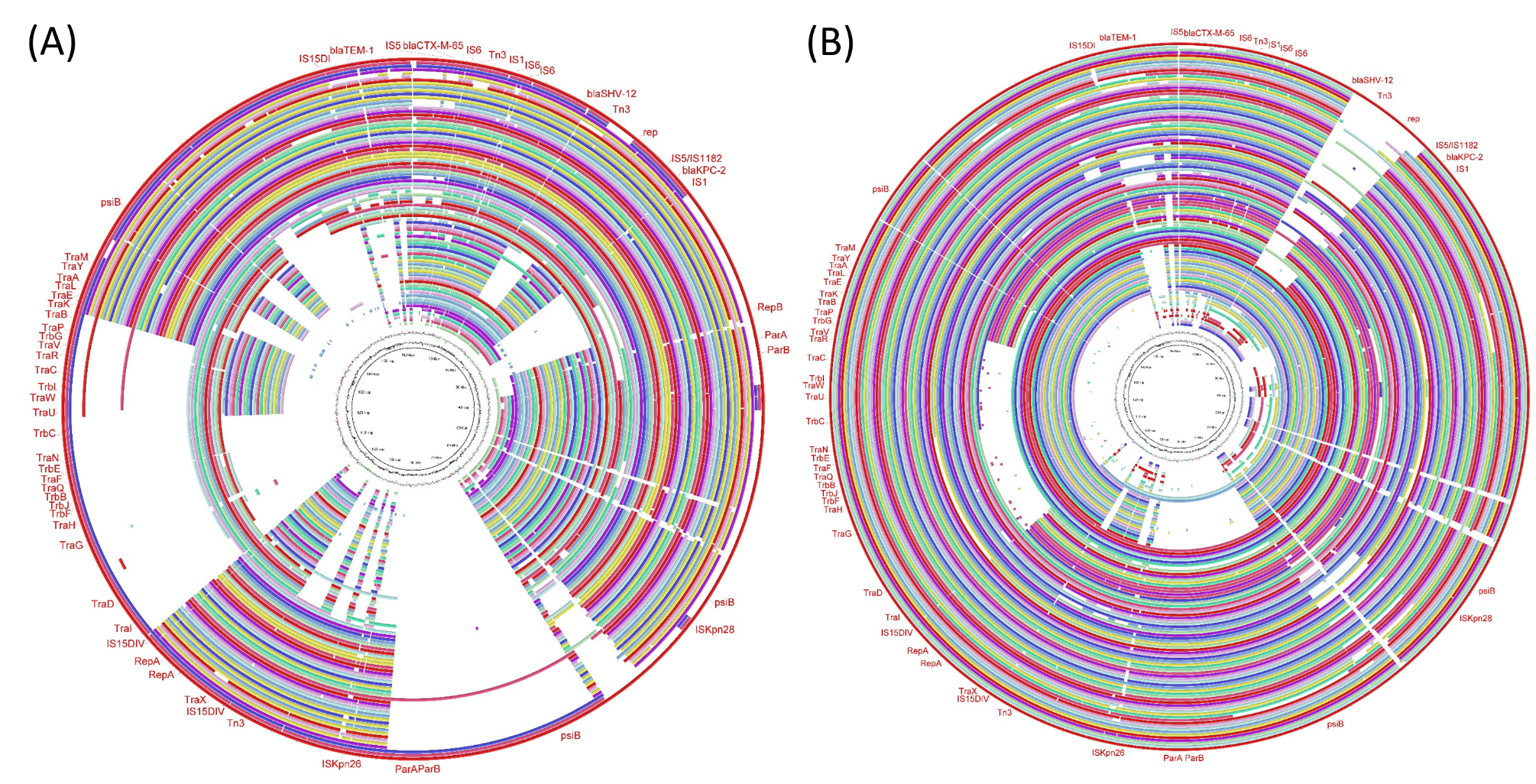


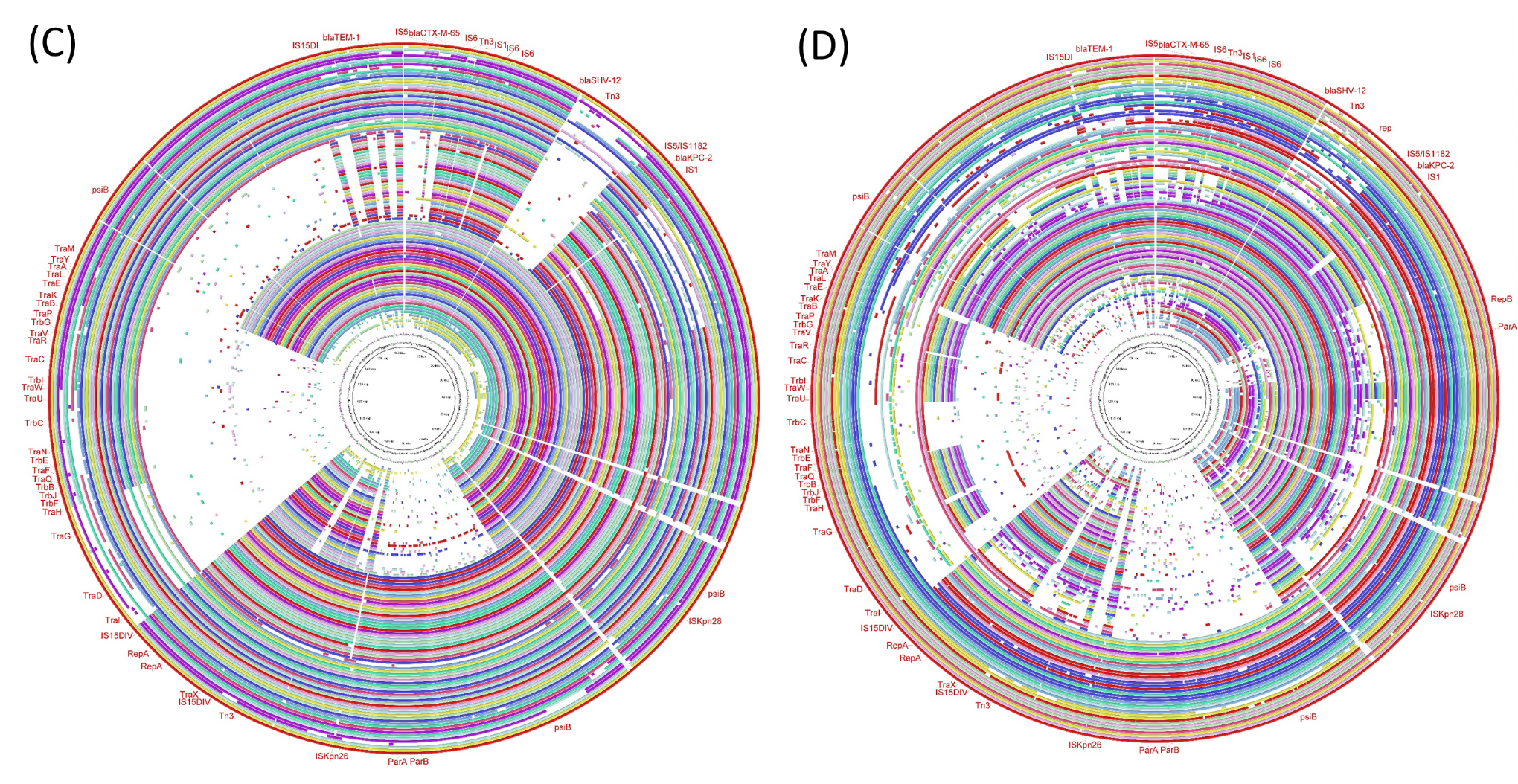


**Supplementary Figure S4. Alignment of *bla*_KPC-2_-bearing plasmids recovered from CRKP strains of three hospitals in China to a *bla*_KPC-2_-bearing plasmid harbored by a *K. pneumoniae* SWU01 strain, pSWU01, isolated from Sichuan China (NZ_CP018455.1) shows that *bla*_KPC-2_-bearing plasmids from CRKP strains from each hospital exhibited a unique pattern which differs from that of the other hospitals.** (A) Alignment of *bla*_KPC-2_-bearing plasmids from CRKP strains of HPPH to the reference plasmid; (B) alignment of virulence plasmids from CRKP strains of WZTH to the reference plasmid; alignment of *bla*_KPC-2_-bearing plasmids from CRKP strains of SAHZJU isolated in 2016 (C) and 2017 (D) to the reference plasmid.
